# Macrocytic anemia induced by selenium deficiency in the course of anorexia nervosa: A case report

**DOI:** 10.1097/MD.0000000000036740

**Published:** 2023-12-22

**Authors:** Ryusei Nishi, Kenichiro Sagiyama, Kazumasa Hamada, Takamasa Fukumoto, Ryuichi Kato, Takako Yamamoto, Yuuki Fuku, Haruka Amitani, Akihiro Asakawa

**Affiliations:** a Department of Psychosomatic Internal Medicine, Kagoshima University Graduate School of Medical and Dental Sciences, Kagoshima, Japan.

**Keywords:** anorexia nervosa, case report, macrocytic anemia, selenium

## Abstract

**Rationale:**

Anorexia nervosa is characterized by an extreme fear of weight gain. Clinicians often prescribe meal replacement shakes if patients are unable or unwilling to consume typical foods. However, these shakes sometimes lack essential micronutrients, such as selenium, which may lead to health risks. Moreover, selenium deficiency induces macrocytic anemia. Herein, we present a case of a patient with anorexia nervosa with macrocytic anemia due to selenium deficiency, which was alleviated by selenium supplementation.

**Patient concerns:**

An 18-year-old female was admitted to our hospital. The patient was diagnosed with anorexia nervosa. Ultimately, she was unable to walk independently because of fatigue and electrolyte disturbances.

**Clinical findings:**

On admission, the height, weight, and body mass index of the patient were 158.5 cm, 27.1 kg, and 10.8, respectively. Our treatment for anorexia nervosa showed relative effectiveness, and the patient’s body weight recovered to 29.2 kg by day 60. However, the mean corpuscular volume increased from day 20, suggesting macrocytic anemia.

**Diagnoses, interventions, and outcomes:**

Despite our vitamin B12 and folic acid supplementation interventions, the mean corpuscular volume continued to rise. On day 60, the patient was diagnosed with selenium deficiency, and selenium administration of 100 μg/day was initiated.

**Outcomes:**

The macrocytic anemia in the patient was alleviated, and treatment for anorexia nervosa was continued in our hospital.

**Lessons:**

To the best of our knowledge, this is the first case of macrocytic anemia induced by selenium deficiency with anorexia nervosa comorbidity, underscoring the importance of selenium supplementation in patients with anorexia nervosa, especially in those with macrocytic anemia.

## 1. Introduction

Anorexia nervosa is an eating disorder characterized by an extreme fear of weight gain. Patients often engage in excessive exercise to reduce weight (restricting subtype) or self-induce vomiting immediately after overeating (binge/purge subtype). Clinicians sometimes prescribe meal replacement shakes if patients are unable or unwilling to consume typical foods. However, these shakes may lack essential micronutrients such as selenium, which can lead to health risks.^[1,2]^ Moreover, selenium deficiency is known to induce macrocytic anemia.^[3]^ Herein, we report a case of anorexia nervosa in a patient suffering from macrocytic anemia due to selenium deficiency, which was alleviated by selenium supplementation.

## 2. Patient information and clinical findings

An 18-year-old female was admitted to our hospital. Written informed consent was obtained from the patient. Earlier, the patient was diagnosed with anorexia nervosa caused by amenorrhea at another hospital 2 years after dieting and was subsequently referred to our department. Despite the clinician’s instructions to gradually increase food intake, the body weight of the patient continued to decrease. Finally, the serum liver enzyme levels of the patient were highly elevated, and she could not walk independently because of fatigue and electrolyte disturbances. The patient was admitted to our department as she desired inpatient care.

On admission, the height, weight, and body mass index of the patient were 158.5 cm, 27.1 kg, and 10.8, respectively. The patient did not eat for the first few days, and calorie requirements were met via a peripherally inserted central venous catheter for a week. Thereafter, an intravenous infusion was administered to maintain a total daily calorie (1000 kcal/day) and the dosage was changed according to the oral intake of the patient.

Our treatment for anorexia nervosa was relatively effective, and body weight gradually increased to 29.2 kg by day 60. However, the mean corpuscular volume (MCV) increased from day 20, suggesting that anemia could not have been caused by iron deficiency alone, which is frequently observed in patients with anorexia nervosa because of restricted dietary intake. Laboratory results during hospitalization are shown in Table 1.

**Table 1:** Laboratory results during hospitalizaion.

Laboratory test	Normal range	Day 1	Day 20	Day 40	Day 60	Day 66	Day 73	Day 83	Day 90
RBC	3.86–4.92 × 10^6^ μL	3.12	1.75	1.93	2.25	2.46	2.76	2.93	3.42
Hb	11.6–14.8 g/dL	10.3	5.7	6.3	7.6	8.4	9.4	9.9	11.5
MCV	83.6–98.2 fL	95.8	95.4	100.5	105.8	103.3	102.2	99.0	96.2
VB12	233–914 pg/mL	–	3670	–	–	–	–	–	–
FA	3.6–12.9 ng/mL	–	13.4	–	–	–	–	–	–
Se	10.0–16.0 μg/dL	–	–	–	6.8	–	11.2	–	–
P	2.7–4.6 mg/dL	2.7	1.9	3.8	4.3	4.7	4.2	3.9	3.4
Ca	8.8–10.1 mg/dL	7.9	7.4	7.5	8.4	8.4	8.7	8.9	9.2
Na	138–145 mmol/L	141	141	144	144	144	142	139	139
K	3.6–4.8 mmol/L	2.9	3.3	3.5	3.5	3.7	3.7	3.9	3.7
Cl	101–108 mmol/L	98	102	109	108	108	106	104	101
TP	6.6–8.1 g/dL	–	3.8	3.9	4.7	5.0	5.7	5.9	6.4
Albumin	4.1–5.1 g/dL	–	2.2	–	2.7	–	–	3.7	–

Ca = calcium, Cl = chloride, FA = folic acid, Hb = hemoglobin, K = potassium, MCV = mean corpuscular volume, Na = sodium, P = phosphorus, Se =selenium, TP = total protein, VB12 = vitamin B12.

## 3. Diagnostic assessment and therapeutic intervention

On day 40, vitamin B12 deficiency was suspected to have caused macrocytic anemia. Consequently, we initiated supplementation with folic acid (10 mg/day) and vitamin B12 (500 μg/day). Despite this intervention, MCV continued to increase. We then reduced the folic acid dose to 5 mg/day, suspecting that an inappropriate initial dose might have been administered because of the imbalanced vitamin B12 and folate levels. Nonetheless, the MCV continued to increase and was 105.8 fL on day 60.

Despite the oral calorie intake of approximately 700 kcal/day, which would generally not suggest a severe micronutrient deficiency, we considered selenium deficiency to be a possible cause. Finally, the patient was diagnosed with selenium deficiency, and selenium administration of 100 μg/day was initiated on day 60. On day 73, selenium levels increased to normal, MCV started to decrease, and hemoglobin levels increased.

## 4. Outcomes

After 20 days of intravenous selenium administration, the MCV returned to normal levels, and selenium supplementation was stopped. The patient continued receiving treatment for anorexia nervosa in our hospital.

## 5. Discussion

Our case strongly suggests that macrocytic anemia in the patient was induced by selenium deficiency, as confirmed by normalization of the patient’s MCV after selenium supplementation. A study on selenium supplementation in anorexia nervosa reported that selenium deficiency is associated with increased severity and suicide risk in anorexia nervosa.^[4]^ To the best of our knowledge, selenium deficiency in patients with anorexia nervosa were diagnosed with white muscle disease, which is an extremely rare condition in humans, has only been reported by Ishihara et al^[5]^ In contrast, Ahmed et al reported the occurrence of macrocytic anemia in adult lambs with white muscle disease.^[6]^ Therefore, the association between macrocytic anemia and anorexia nervosa because of the selenium deficiency appears plausible. However, Ishihara et al^[5]^ did not report macrocytic anemia in their patients. Therefore, this is the first case of macrocytic anemia induced by selenium deficiency with comorbid anorexia nervosa.

The mechanism underlying macrocytic anemia induced by selenium deficiency is not fully understood. However, selenium prevents hemolytic anemia by protecting the cell membrane from hemolysis by modulating oxidation–reduction reactions. Therefore, selenium antioxidation may play a key role in preventing macrocytic anemia. Notably, radical-related stress scavenger knockout mice exhibit a macrocytic anemia phenotype.^[7]^ Nonetheless, further research is required to elucidate the role of selenium in macrocytic anemia. Our case highlights the importance of selenium supplementation in patients with anorexia nervosa, especially in those with macrocytic anemia.

## Author contributions

**Conceptualization:** Ryusei Nishi, Kenichiro Sagiyama, Akihiro Asakawa.

**Data curation:** Ryusei Nishi, Kenichiro Sagiyama.

**Investigation:** Ryusei Nishi, Kazumasa Hamada, Takamasa Fukumoto, Ryuichi Kato, Takako Yamamoto, Yuuki Fuku, Haruka Amitani.

**Supervision:** Haruka Amitani, Akihiro Asakawa.

**Writing – original draft:** Ryusei Nishi.

**Writing – review & editing:** Ryusei Nishi, Kenichiro Sagiyama, Kazumasa Hamada, Takamasa Fukumoto, Ryuichi Kato, Takako Yamamoto, Yuuki Fuku, Haruka Amitani, Akihiro Asakawa.
